# A Novel Statistical Algorithm for Gene Expression Analysis Helps Differentiate Pregnane X Receptor-Dependent and Independent Mechanisms of Toxicity

**DOI:** 10.1371/journal.pone.0015595

**Published:** 2010-12-21

**Authors:** M. Ann Mongan, Robert T. Dunn, Steven Vonderfecht, Nancy Everds, Guang Chen, Cheng Su, Marnie Higgins-Garn, Yuan Chen, Cynthia A. Afshari, Toni L. Williamson, Linda Carlock, Christopher DiPalma, Suzanne Moss, Jeanine Bussiere, Charles Qualls, Yudong D. He, Hisham K. Hamadeh

**Affiliations:** 1 Comparative Biology and Safety Sciences, Amgen Inc., Thousand Oaks, California, United States of America; 2 Research and Translational Sciences Biostatistics, Amgen Inc., Seattle, Washington, United States of America; University of North Carolina at Charlotte, United States of America

## Abstract

Genome-wide gene expression profiling has become standard for assessing potential liabilities as well as for elucidating mechanisms of toxicity of drug candidates under development. Analysis of microarray data is often challenging due to the lack of a statistical model that is amenable to biological variation in a small number of samples. Here we present a novel non-parametric algorithm that requires minimal assumptions about the data distribution. Our method for determining differential expression consists of two steps: *1*) We apply a nominal threshold on fold change and platform p-value to designate whether a gene is differentially expressed in each treated and control sample relative to the averaged control pool, and *2*) We compared the number of samples satisfying criteria in step 1 between the treated and control groups to estimate the statistical significance based on a null distribution established by sample permutations. The method captures group effect without being too sensitive to anomalies as it allows tolerance for potential non-responders in the treatment group and outliers in the control group. Performance and results of this method were compared with the Significant Analysis of Microarrays (SAM) method. These two methods were applied to investigate hepatic transcriptional responses of wild-type (PXR^+/+^) and pregnane X receptor-knockout (PXR^−/−^) mice after 96 h exposure to CMP013, an inhibitor of β-secretase (β-site of amyloid precursor protein cleaving enzyme 1 or BACE1). Our results showed that CMP013 led to transcriptional changes in hallmark PXR-regulated genes and induced a cascade of gene expression changes that explained the hepatomegaly observed only in PXR^+/+^ animals. Comparison of concordant expression changes between PXR^+/+^ and PXR^−/−^ mice also suggested a PXR-independent association between CMP013 and perturbations to cellular stress, lipid metabolism, and biliary transport.

## Introduction

Microarrays are now the preferred technology in many biological applications ranging from functional characterization of genes and pathways to classification of disease signatures for diagnostic and prognostic purposes. Within the field of toxicogenomics, genome-wide gene expression data have been widely used to assess potential toxicity as well as to elucidate mechanisms of toxicity of drug candidates [1]–[4]. The growing number of applications and wide adoption of microarray data have in turn fueled the development of analysis methods devised to extract information from these datasets [5]. While earlier studies were often complicated by technical inconsistencies, microarray data have become significantly more reliable and reproducible [6], [7]. In fact, analysis of reference mRNA obtained from mixed rat tissues processed over a multi-year period by Amgen and several external facilities has consistently shown relatively high sensitivity and specificity [8], [9]. However, even with highly improved technology, the microarray community continues to struggle with the analysis, interpretation, and extraction of biologically relevant knowledge from the large volume of expression measurements. Much work has been invested in developing models and algorithms for these purposes and their levels of complexity have tended to increase over time. Unfortunately, however, the increased level of algorithmic complexity does not always translate to improved biological understanding [10]. In particular, many model-driven methods often assume certain distributions for the data that are either not true or not easily verifiable. Furthermore, while most existing statistical models perform well with simulated data, they often are too sensitive to what is generally considered an acceptable level of biological variation.

Our goal here is to devise a method that requires fewer assumptions about intensity distribution of genes and therefore can be described with an intuitive mathematical model. The method is meant to capture group effects without being too sensitive to anomalies in a small subset of subjects. Such tolerance is necessary because biological variation is typically large due to uncontrollable variables resulting both from inherent heterogeneity and technical procedures during the course of experiments. Therefore, it may not be desirable to penalize large variations in the amplitude of gene expression as long as the changes relative to the control group are in the same direction across the majority of animals. Our approach leverages the platform p-value [11] and fold change cutoffs to designate whether changes in a gene constitute biologically differential expression between each treated sample relative to the vehicle-control pool. The significance of the group effect for the gene is then estimated by comparing the number of changed samples observed between the two groups. In particular, the algorithm involves two steps:

1. A nominal threshold of 1.25 fold change and platform p-value [11] of 0.1 were used to designate whether a gene displays differential expression in each treated and control sample relative to the averaged control pool.
2. False discovery rate was estimated based on the probability of encountering the observed number of differentially expressed samples in the treated group, given the total number of observed differentially expressed samples in both groups, under the empirically determined distribution derived from the null hypothesis that the differentially expressed samples are equally distributed in both groups.

**Figure 1:**
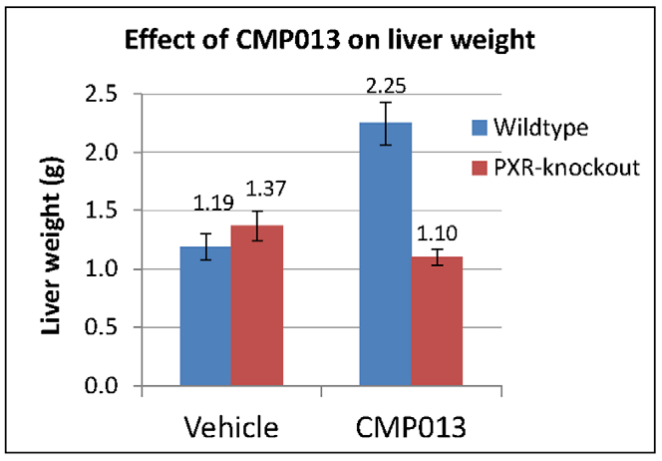
Effect of CMP013 on liver weight of wild type and PXR-knockout mice. Wild type mice showed similar liver weight increase as previously observed in Sprague Dawley rats; such increase was absent in the knockout strain.

Thresholds on fold change and p-values were set to values we believed would likely translate to biological significance from our experience with similar toxicology studies. Performance of this method was compared with the popular microarray analysis method Significant Analysis of Microarrays (SAM) [12]. The two methods were applied to investigate the role of pregnane X receptor (PXR) in hepatic toxicities induced by CMP013, a small molecule inhibitor of β-secretase (BACE1) enzyme. BACE1, the first of the two proteases that cleaves amyloid precursor protein, is believed to be a prime drug target for Alzheimer's disease [13]–[15]. Early toxicology screening with CMP013 in rats revealed prominent effects in the liver including hepatomegaly (liver weight nearly 2x above control) with histological correlates of increased mitotic figures, vacuolation, and hepatocellular hypertrophy. *In vitro* data suggested that CMP013 might be an agonist for PXR and we hypothesized that this nuclear receptor was at least partially responsible for the potent hepatic effects noted in 4-day rat toxicology studies. To further evaluate the role of PXR in mediating mechanisms of toxicity by CMP013, a subsequent 4-day toxicology study with CMP013 was carried out with wild type (C57Bl/6) and PXR-knockout (C57Bl/6NTac) mice (Table 1) to confirm that mice respond to CMP013 in a manner similar to rats. Following confirmation of CMP013-mediated hepatic effects in C57Bl/6 mice (Figure 1), gene expression data was generated from liver tissue of these animals for mechanistic investigation. Specifically, the data from this knock-out study allowed us to differentiate PXR-dependent and independent mechanisms of this BACE1 inhibitor in mediating the observed hepatotoxic effects.

**Table 1:** Study Design.

Strain	Test Article	Dose level (mg/kg/day)	Dose volume (mL/kg)	Concentration (mg/mL)
C57Bl/6 (WT)	Vehicle^a^	0	10	0
C57Bl/6 (WT)	CMP013	150	10	15
C57Bl/6NTac (PXR-KO)	Vehicle^a^	0	10	0
C57Bl/6NTac (PXR-KO)	CMP013	150	10	15

All animals were 9-week old males at initiation of treatment. Mice were dosed via oral gavage every 24 h and euthanized at 96 h. Each of the following groups contains 5 animals.

a 2% HPMC/1% Tween 80 in DI water, pH 2.2. adjusted with methanesulfonic acid.

## Materials and Methods

### Ethics Statement

All animals were handled in strict accordance with good animal practice as defined by the relevant national and local animal welfare bodies, and all animal work was approved by the Amgen's Institutional Animal Care and Use Committee under IACUC protocol 2008-00174.

### *In vivo* study

Two groups of mice, C57Bl/6 (PXR^+/+^) and PXR-knockout C57Bl/6NTac (PXR^−/−^), were administered by oral gavage either CMP013 or vehicle (2% HPMC/1% Tween 80 in DI water, pH 2.2. adjusted with methanesulfonic acid) according to dose levels outlined in Table 1. Food (irradiated Harlan Teklad rodent maintenance diet) and water were available *ad libitum* during the study except for the last 3–4 h prior to necropsy during which animals were fasted and only water was available. Actual food intake and water consumption were not monitored for individual animals during the course of study. At 96 h, all animals were euthanized via CO_2_ asphyxiation followed by exsanguination. Liver samples were promptly collected and frozen until ready for RNA extraction.

### RNA Isolation

Total RNA was isolated from pieces of mouse liver according to the RNeasy extraction procedure (Qiagen, Valencia, CA). Tissues were homogenized in QIAzol lysis buffer using the GenoGrinder 2000 homogenizer (SPEX SamplePrep, Metuchen, NJ). Samples were processed on the Qiagen BioRobot Universal system according to the manufacturer's instructions. An on-column DNase digestion was performed to remove any residual genomic DNA contamination. RNA concentration and yield were measured spectrophotometrically using the Nanodrop instrument. Quality of the nucleic acid samples was evaluated with the RNA 6000 Nano chip kit (Agilent Technologies, Expert software). Quality of samples was verified with distinct ribosomal 18S & 28S peaks, low baseline, and high RIN values (PXR^+/+^: 9.3-10; PXR^−/−^: 8.7-9.6).

### Gene expression data generation

Liver RNA from individual mice was profiled separately on the Affymetrix GeneChip® platform without technical replication. Microarray profiling was performed by Cogenics (Morrisville, NC). Briefly, 1 µg of total RNA was reverse transcribed to double stranded cDNA with the BioarrayTM Single-Round RNA Amplification and Labeling Kit and biotinylated cRNA was generated using the BioArray™ HighYield™ RNA Transcript Labeling Kit (Enzo Life Sciences, Farmingdale, NY). For each sample, 10 µg of biotinylated cRNA spiked with hybridization controls (bioB, bioC, bioD and cre) was hybridized to an Affymetrix Mouse 430_2 microarray for 16 h at 45°C. Following hybridization, arrays were washed and stained in an Affymetrix GeneChip Fluidics Station and scanned with a GeneChip® Scanner 3000 (Affymetrix, Santa Clara, CA). Quality checks and data analyses were carried out using Affymetrix GeneChip Operating Software (GCOS) and Quality Reporter. All data were MIAME compliant and raw data (cel files) have been deposited to a MIAME compliant database, GEO, accession GSE23780.

### Gene expression analysis

#### Analysis with the proposed method

Log-ratios of gene expression data and associated platform p-values [11] were generated in the Rosetta Resolver System (Rosetta Biosoftware, Seattle, WA, version 7.2) for all profiles relative to strain-matched vehicle-treated controls. For each Affymetrix sequence (or probe set), log-ratio was defined to be the log_10_ of the intensity ratio of each animal (either treated with vehicle or CMP013) to the mean intensity of that sequence across the five profiles in the corresponding vehicle control group. To identify differentially expressed sequences due to CMP013 treatment, we carried out a two-step non-parametric statistical analysis, which was applied to data from wild type (WT) and PXR-knockout (PXR-KO) mice separately. This analysis was performed in the programming language R.

**Figure 2:**
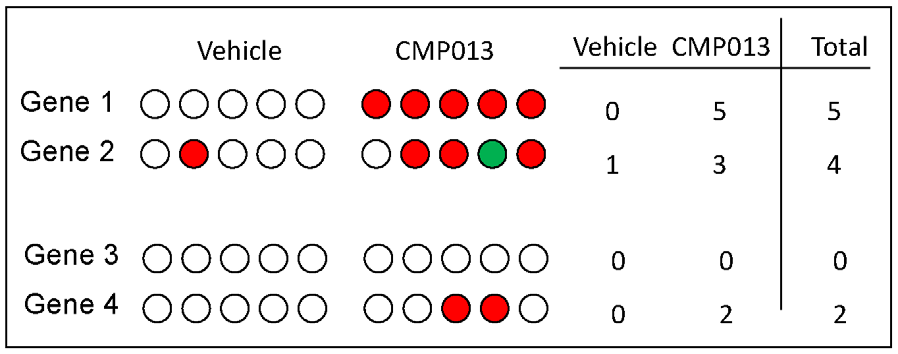
Counting procedure defined by step 1. In this diagram, colored circles represent profiles (animals) in which a sequence *i* satisfies |fold change|≥1.25 and platform p-value ≤0.1, while open circles represent samples that do not. A red circle symbolizes up-regulation and a green circle symbolizes down-regulation. The counting step simply records a sum of the number of profiles showing changed in the same direction.

Step 1: Counting the number of samples in which a sequence i showed differential expression based on platform p-value and fold change. For each sequence, we identified the number of samples that satisfy |log-ratio|≥0.097 (equivalent to a fold change cutoff of 1.25) and platform p-value ≤0.1. The number of animals passing this criterion was counted separately for the vehicle and the CMP013-treated groups (Equation 1)
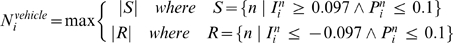
(1)where 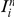 is the log-ratio intensity of gene *i* measured in animal *n,* 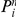
is the associated p-value of gene *i*, and 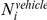 represents the number of animals satisfying the above conditions for sequence *i* in the vehicle group. 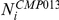 is defined similarly for animals in the treatment group. |S| represents the number of animals in which sequence *i* was potentially *up-regulated* relative to the control pool, while |R| represents the number of animals in which that sequence was potentially -*down-regulated*. For each of WT or PXR-KO dataset, this step produced two vectors of length equal the number of sequences on the GeneChip® (Figure 2). In cases where |S| = |R|, i.e., the genes show increased and decreased expression in equal number of animals, such expression change was designated non-interpretable and step 2 was not necessary.

Step 2: Estimate of statistical significance for group difference between treatment and vehicle. A gene is identified as having significantly altered expression by CMP013 treatment if a significantly greater number of animals in the treatment group satisfy the criteria in step 1 as compared to those in the control group. In other words, we need to evaluate the probability of getting a pairing of (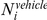,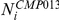) by chance. The null hypothesis for a sequence *i* is that, for a given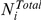 = *t*, the numbers of animals that satisfied conditions in step 1 are equally distributed between the vehicle (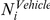) and treated group (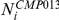) (Equations 2–4). The values for *t* range from 0–9 because conditions for significance are set relative to the mean of the control group and thus the maximal significant samples from this group is 4.
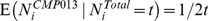
(2)

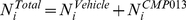
(3)

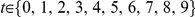



A sequence *i* is deemed significantly differentially expressed by CMP013 if 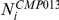 is equal or larger than a cutoff, which is determined by controlling the false discovery rate (FDR). A null distribution was created by permuting the profile labels among vehicle and treated groups 252 times (exhaustive combinations) and re-computing step 1 on this randomized data. For a given number of significant samples *j* in treated group, with 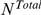 = *t*, the FDR is given by Equation 4.
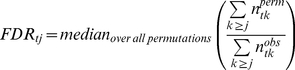
(4)


The denominator in the fraction represents the number of genes that would be considered significant from observed data; the numerator represents the number of genes considered significant from permutation. Sequences with *FDR_tj_*≤0.05 were considered statistically significant (Table 2).

**Table 2:** False discovery rate (FDR) estimated based on wild-type (A) and PXR-knockout (B) data.

A	WT	0	1	2	3	4	5
	**0**	1					
	**1**	1	0.558				
	**2**	1	0.827	0.227			
	**3**	1	0.925	0.500	0.058		
	**4**	1	0.975	0.781	0.183	0.001	
	**5**	1	0.993	0.983	0.484	0.003	0
	**6**	1	0.993	0.990	0.692	0.044	0
	**7**	1	0.980	0.980	0.843	0.144	0
	**8**	1	0.947	0.947	0.842	0.333	0
	**9**	1	1	1	1	0.5	0

B	PXR-KO	0	1	2	3	4	5
	**0**	1					
	**1**	1	0.632				
	**2**	1	0.831	0.225			
	**3**	1	0.897	0.473	0.028		
	**4**	1	0.938	0.808	0.080	0.001	
	**5**	1	0.973	0.948	0.404	0.005	0
	**6**	1	0.979	0.973	0.642	0.046	0
	**7**	1	0.977	0.977	0.826	0.217	0
	**8**	1	1	1	0.923	0.385	0
	**9**	1	1	1	1	0.5	0

Each value F*_ij_* in the table indicates the FDR for a gene found to be differentially expressed (based on fold change and platform p-value cutoffs) in *j* samples of the CMP013 treatment group out of *i* samples that are differentially expressed in both groups. Underlined values correspond to cases where the genes would be considered statistically significant at FDR≤0.05. FDR = 0 corresponds to events that were not observed in permutated data.

#### Analysis with SAM

SAM analysis was carried out using the method *sam* in the Bioconductor [16] package *siggenes*. The input was a data frame containing log_2_ intensity of 10 profiles (vehicle and treatment) in either the WT or PXR-KO dataset. The class label was a vector of 10 elements, assigning zeros for vehicle and ones for treatment profiles. The default method *d.stat* (a modified t-test) as defined by Tusher *et. al.* [12] was applied as the test statistics. The output of *sam* was a table containing a list of 10 delta cutoffs, the number of genes deemed statistically significant at these cutoffs and associated FDR. We used this table to fine tune delta values so that the final FDR was <10% below the desired FDR of 0.05. The resulting gene lists for both WT and PXR-KO data were used for pathway analysis.

### Pathway Analysis

Pathways associated with differentially expressed genes identified by our proposed algorithm and SAM were analyzed using the Tox Analysis function in IPA (Ingenuity Systems, Redwood City, CA) [Application version: 8.0, Build: 82437; Content version: 2602]. Canonical pathways and tox list (list of genes associated with toxicities as determined by IPA) with p-value ≤0.05 were deemed significantly perturbed pathways.

## Results

### Identification of differential expressed genes due to CMP013 treatments

#### Results obtained with current method

**Figure 3:**
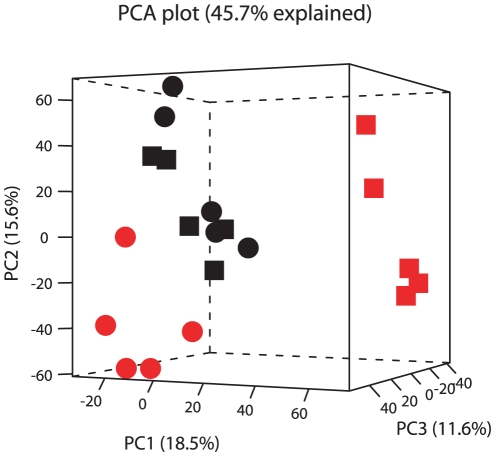
Principle component analysis. The first three principle components are based on log_2_ intensity and shown as four groups: (□) WT, (O) PXR-KO, black: vehicle, red: CMP013 treatment.

The Mouse Affymetrix 430_2 GeneChip® contains 45,037 probe sets, which we refer to as sequences in this paper. The principle component analysis (46% explained, Figure 3) of these sequences, based on quantile-normalized intensity data, showed that separation was observed for animals treated with vehicle vs. CMP013. In fact, the effect of CMP013 treatment appeared much larger than the effect of knocking out PXR, highlighting the fact that this compound modulated a relatively large number of genes in mouse liver.

**Figure 4:**
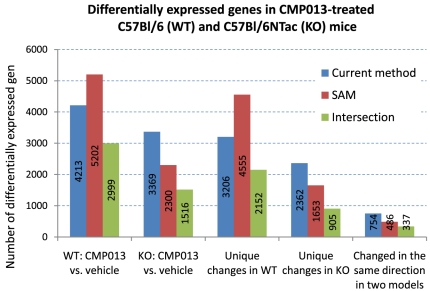
Differentially expressed genes in CMP013-treated C57Bl/6 (WT) and C57Bl6NTac (PXR-KO) mice. “CMP013 vs. vehicle” represents genes identified as differentially expressed due to CMP013 treatment; “Changed in WT model only” represent genes that are likely mediated by PXR in response to compound treatment; and “Changed in the same direction in two models” represents sequences that modulated by the compound independent of PXR regulation.

Log-ratio data and associated platform p-values for all sequences were generated in Rosetta Resolver (version 7.2). Results with the current method showed that CMP013 led to 4,213 and 3,369 differentially expressed sequences in the livers of WT and PXR-KO mice, respectively (Figure 4, Data S1). In the WT model, the majority of sequences differentially expressed by CMP013 were changed in all five animals in the treatment group. In the PXR-KO model, however, the majority of differentially expressed sequences were changed in only three animals in the treatment group. This suggested that the transcriptional response to CMP013 in PXR-KO mice was less homogeneous than that in WT mice. We speculated that the difference in group behaviors between the knockout and WT strains was a result of different compensatory mechanisms each knockout animal developed to compensate for the absence of PXR regulation. There was not sufficiently strong evidence to support this hypothesis in the current study, but we are investigating mouse strains with knockout of other nuclear receptors to determine if similar behaviors are exhibited. Nevertheless, our method is particularly well-suited for studies in both preclinical and clinical settings where subject-to-subject variation is relatively large. Overall, we identified 20% more differentially expressed sequences due to CMP013 treatment in the WT model as compared to the PXR-KO model. In addition, most of these sequences appeared to be strain-specific: 76% of sequences changed in the WT model were found only in this model and 70% of sequences changed in the PXR-KO model were unique to that model. About 18% of the total number of differentially expressed sequences in WT mice were similarly modulated (either up-regulation or down-regulation) in PXR-KO mice; these sequences and associated genes were considered to represent PXR-independent effects.

#### Comparison with results obtained with SAM

**Figure 5:**
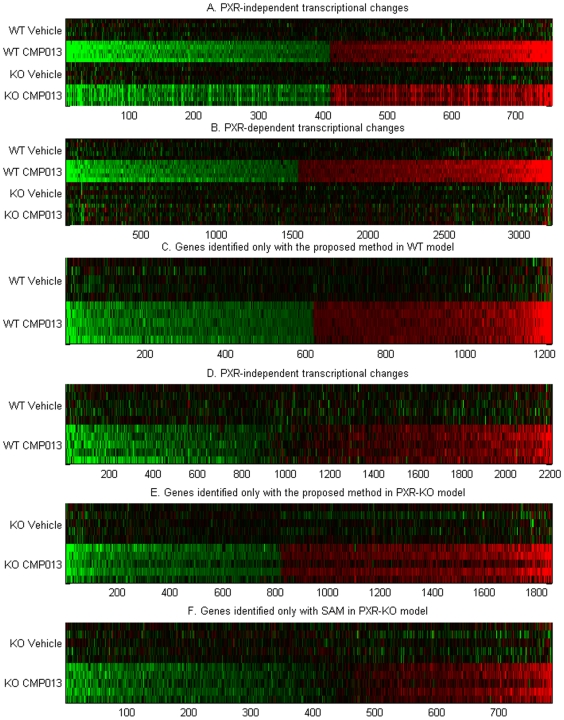
Heat maps of differentially expressed genes identified by the proposed method and SAM. Panels A and B show genes that transcriptionally respond to CMP013 treatment in a PXR-dependent and independent manners. Panels C and E show the subset of genes identified only by our proposed method and panels D and F show genes identified only by SAM.

SAM returned 5,202 differential expressed sequences in CMP013-exposed WT mice and 2,300 sequences in the PXR-KO mice (Figure 4, Data S1). That is, at a false discovery rate of 5%, SAM identified more sequences than our proposed method in the WT model, and we identified more sequences than SAM in the PXR-KO model. In designing our analysis, we wanted to give considerations to both biological and statistical significance. The key difference between the proposed method and SAM is that our method used a threshold approach to determine biological significance and group effect to determine statistical significance determination. In addition, we did not penalize large variation of changes within the same treatment group as long as the changes were in the same direction. As a result, though SAM returned approximately 20% more statistically significant sequences than our method in the WT model; the vast majority of them (85%) did not satisfy a fold change cutoff of 1.25. While such changes were sufficiently homogenous among animals in the same treatment group to achieve statistical significance; the increase or decrease might be too small to warrant biological difference. On the other hand, the proposed method identified 1,214 additional sequences that were not returned by SAM. These sequences were more likely to represent significant biological differences because their expression changes, though varied from animal to animal, were in the same direction. Furthermore, since there were more non-responders in the PXR-KO group, the current method was more sensitive than SAM and was able to return many sequences which did not express large differential expression in 1–2 animals. In other words, the SAM method is less sensitive when group behavior is less consistent. Heat maps of sequences identified only by the current method or SAM further illustrate the differences in results of the two methods (Figure 5 C–F). Panels C and E show that sequences found by our method have clear differential expression (due to fold change cutoff) as compared to the control group even when the magnitude of difference is relatively small. In contrast, panels D and F show that a portion of sequences identified by SAM do not display visible differential expression. That is, sequences identified only by our method cover a larger ranges of (unidirectional) fold changes, whereas those identified only with SAM were likely sequences that had less than 1.25 fold change but were consistent among all five animals in the treatment group. As noted earlier, sequences in this latter category could appropriately be considered to have statistically significant differences, but such differences may not be sufficient to constitute biological significance. It is, of course, up to the investigator to decide how much change would constitute biological difference, and the approach presented in this paper is designed to be amenable to such modification.

### Transcriptional effects of BACE1 inhibitor CMP013

We grouped CMP013-modulated sequences into two categories: *A*) sequences uniquely changed in the WT model, i.e., PXR-dependent effects and *B*) sequences showing concordant changes between the WT and PXR-KO models, i.e., PXR-independent response to CMP013 treatment. Sequences in group *A* were found to be involved in PXR-mediated pathways, as their expression was modulated only in WT mice and unchanged in the knockout model (Figure 5A). Examination of this group of pathways in relation to histopathology findings provided a clearer understanding of PXR's role in the dramatic hepatocellular hypertrophy observed in WT mice. Sequences in group B reflected properties of the compound that did not depend on the presence of PXR (Figure 5B). These groups of sequences were transferred to IPA where they were mapped to known genes and associated pathways. It should be noted, however, that only about 50% of sequences were mapped to annotated genes and pathways in IPA, and thus our interpretation of these data was limited by this annotation. When there were multiple sequences mapped to the same gene symbol, the sequence with the largest fold change was assigned to that gene.

#### PXR-dependent transcriptional effects

Parallel treatment of wild type and PXR-KO mice with CMP013 allowed us to elucidate transcriptional responses that were dependent on PXR regulation. Knockout of the nuclear hormone receptor PXR completely prevented the CMP013-induced increases in liver weights, hepatocellular hypertrophy and mitotic activity. Comparison of gene expression data obtained from two groups of animals provided molecular evidence for many of these changes. In particular, CMP013 increased the expression of 3,206 genes exclusively in WT mice, many of which were indicative of PXR/CAR agonism properties of this compound. In particular, there was increased expression of hallmark P450 genes including Cyp3a4 (4.3 fold) and Cyp2b6 (88 fold). We additionally observed induction of genes encoding phase 1 and 2 enzymes such as aldehyde dehydrogenase (1a1, 1a7, 1b1, and 18a1, 1.5-2 fold), glutathione S-transferase alpha (Gsta4, 4.5 fold; Gsta5, 11 fold), glutathione S-transferase mu (members 1, 3, 4, 5 and 6; 1.3-11 fold), and UDP glucuronosyltransferase (Ugt2b10 and Ugt2b15, ∼2 fold). Besides these xenobiotic metabolism genes, PXR regulation also had roles in many hepatotoxicity-related cellular processes with the notable ones described below.

Consistent with the hepatomegaly and liver weight increase observations (Figure 1), altered expression of genes involved in G1/S and G2/M cell cycle check points was observed in WT mice. Increased expression of cyclin-dependent kinase 1 (Cdk1, 3.3 fold) and cyclin D1 (Ccnd1, 2 fold) suggested that CMP013 enabled a growth-induced condition, which allowed the cells to overcome the G1/S checkpoint. Complexes involved Ccnd1 have been reported to phosphorylate the tumor suppressor retinoblastoma, which in turn relieved the inhibition on the transcription factor E2f [17], [18]. Consistent with this report, we observed 2-3 fold increased expression of retinoblastoma-binding proteins (Rbll), E2f3, and E2f6. The second checkpoint is located at the end of G2 phase and the beginning of the M phase, which ensures the cell's readiness for mitosis. Though many proteins involved in this process were regulated by post-translational modification, transcriptional changes were observable for Chk1 checkpoint homolog (Chek1, 2.9 fold) and cyclin B1 and B2 (Ccnb1, 2.2 fold; Ccnb2, 4 fold). The induction of these genes underscored the role of PXR in cellular proliferation as a likely contributor to the profound liver weight increase observed in WT but not PXR-KO mice.

The protein ubiquitination pathway plays a major role in the degradation of regulatory proteins involved in cell cycle, apoptosis, and a variety of signaling processes in the cell. In WT mice treated with CMP013, we observed an increase in proteasomal degradation evidenced by the transcriptional changes of genes encoding these enzymes. The degradation of proteins via the protein ubiquitination pathway begins with the conjugation of multiple ubiquitin (Ub) moieties to the target protein followed by the degradation of the polyubiquitinated protein by the 26S proteasome complex. Though expression changes in Ub-activating enzyme (E1) were not observed, altered expression was noted for numerous Ub-conjugating enzymes E2 (2f, 2k, 2l, 2m and 2v increased ∼1.5 fold; 2b, 2d, 2e, 2i, and 2n decreased 1.3-2 fold), Ub protein ligase E3 (Ube3a, 1.6 fold increase), and ubiquitination factors E4 (Ube4a and 4b, ∼1.5 fold), which participated in multi-Ub chain assembly. The polyubiquitin chain was recognized by the multi-catalytic proteasome complex which completed the proteolysis process. Transcriptional increase of genes making up the proteasome complex was accordingly observed (Psma, Psmb, Psmc, Psmd, and Psme, 1.3-2 fold).

Significant up-regulation of aminoacyl-tRNA biosysnthesis was also observed exclusively in WT mice. Approximately two fold transcriptional increase was noted for tRNA of most amino acids including ala-tRNA synthetase (Aars and Aarsd1), arg tRNA synthetase (Rars), asp tRNA synthetase (Nars), glu-tRNA synthetase (Ears2), ile-tRNA synthetase (Iars), leu-tRNA synthetase (Lars), lys-tRNA synthetase (Kars), phe-tRNA synthetase (Farsa), and trp-tRNA synthetase (Wars) The increased transcription in this process together with similar increases in the ubiquitination pathway indicated that CMP013 led to a high rate of protein turnover in the cells, likely associated with cell division.

Furthermore, CMP013 was associated with transcriptional modulation of genes that were suggestive of an activation of the NFκB signaling pathway in WT mice treated with CMP013. In particular, we observed increased expression of TGFα (1.4 fold), toll-interleukin 1 receptor domain containing adaptor protein (Tirap, 1.3 fold), TNF receptor-associated factor 3 (Traf3, 2 fold), eukaryotic translation initiation factor 2-alpha kinase (Eif2ak2, 1.7 fold), and inhibitor of kappa light polypeptide gene enhancer (Ikbkg, 1.7 fold). The activation of this signaling pathway was suggestive of an inflammatory response and was consistent with the severity and frequency of the inflammatory infiltrates observed in this group of mice.

#### PXR-independent transcriptional effects

We identified 754 sequences that were perturbed in the same direction in both WT and PXR-KO mice treated with CMP013. Genes associated with these sequences were mapped to five biological processes. In addition to this shared gene set, we observed groups of differentially expressed genes that were unique in each model of mice, but were mapped to these same five pathways (Table 3).

**Table 3:** Comparison of differentially expressed genes identified by the proposed method and SAM.

	Wild type		PXR-KO	
Gene	Proposed Method	SAM	Proposed Method	SAM
**Fatty acid metabolism**				
ACAA1	−1.368	−1.368	−1.619	−1.619
ACAA1B	−1.337	−1.337	−1.642	
ACAA2	−1.318			
ACAD8	−1.545	−1.545	−1.622	−1.485
ACADS	−1.411		−1.427	
ACADSB	−2.616	−2.616		−1.856
ACOX3			−1.382	−1.382
ACSL1	−1.451	−2.093	−2.482	−2.482
CPT1A	−1.402	−1.339		
EHHADH			−1.332	
**Cholesterol biosynthesis**				
DHCR7	1.692	1.692		
FDFT1	2.498	1.862	1.345	1.345
FDPS	1.366			
HMGCR	2.631	2.631	1.55	
HMGCS1	10.515	10.515	2.985	
IDI1	1.544	1.445	1.815	1.815
LSS	2.303	2.303		
MVD	5.342	5.342		
MVK	2.633	2.633	2.968	2.968
SQLE	1.945	1.945	1.242	
**Oxidative Stress**				
DNAJA2			1.439	
DNAJB9	2.367	2.367	1.962	1.962
DNAJC2	1.965	1.965	1.533	
FMO1			1.547	1.547
GPX2	3.464	3.464		
GSR	3.065	3.065		
HMOX1			2.848	2.848
KEAP1	1.445	1.445	1.268	
NQO1	2.392	2.392	1.741	1.741
PRDX1			1.35	
TXN	1.413	1.36		
TXNRD1			1.423	
**Endoplasmic reticulum signaling**				
ATF4			1.64	
ATF6	1.308		1.811	1.811
EIF2AK3	1.416		1.61	1.61
MAPK8	1.557		1.686	
MBTPS2	1.369		1.663	
**Bile acid signaling**				
ABCB1	3.761	3.177	2.89	2.89
ABCC2	1.927	1.927		
ABCC3	2.432	2.432	1.325	1.325
CYP7B1			−1.876	−1.876
CYP8B1			−1.933	−1.933
SLCO1A2	11.494		7.756	

Below are five biological processes and associated genes that were commonly modulated in both WT and PXR-KO mice after 96 h treatment with CMP013. Values in each row are averaged fold change of the gene across all five animals in the treatment group. Fold change values of a gene may differ between the proposed method and SAM if each method identifies a different Affymetrix sequence corresponding to the same gene as significant.

A notable common response to CMP013 between WT and PXR-KO mice was the down-regulation of genes involved in fatty acid metabolism. In particular, decreased expression was observed for key enzymes in β-oxidation including acyl-CoA dehydrogenase (Acads, -1.4 fold in both models), which catalyzes the initial step in each cycle of β-oxidation; and acetyl-CoA acyltransferases (Acaa1 and Acaa1b, both genes: -1.3 fold in WT, -1.6 fold in PXR-KO), which catalyze the final step of the β-oxidation cycle. We additionally observed transcriptional decrease in isoforms of these genes as well as related genes in fatty acid metabolism; these genes were unique to each mouse model. In the WT model, decreased expression of -1.3-3 fold was noted for carnitine palmitoyltranferase (Cpt1a, -1.4 fold), acyl-CoA dehydrogenase, short and branched chain (Acadsb, -2.6 fold), and acetyl-CoA acyltranferase 2 (Acaa2, -1.3 fold). In the PXR-KO model, decreased expression of -1.3-2 fold was observed for acyl-CoA synthetase (Acad8, -1.6 fold), acyl-CoA oxidase 3 (Acox3, -1.4 fold), and enoyl-CoA hydratase (Ehhadh, -1.3 fold). Collectively, these data suggested that treatment with CMP013 was associated with functional perturbation of mitochondrial β-oxidation and that this effect did not necessitate PXR mediation. Additionally, CMP013 led to increased biosynthesis of cholesterol in both mouse strains. Evidence of this increase included the ∼2 fold increased expression of the rate limiting enzyme HMG-CoA reducatase (Hmgcr) and numerous genes in this pathway such as mevalonate kinase (Mvk), squalene epoxidase (Sqle), isopentenyl-diphosphate delta isomerase 1 (Idi1), and farnesyl diphosphate farnesyl transferase 1 (Fdft1). Similar increases in expression were noted for other genes in the pathway, though the changes were only observed in WT mice: lanosterol synthase (Lss), farnesyl diphosphate synthase (Fdps) and 7-dehydrocholesterol reductase (Dhcr7). Besides cholesterol, metabolism of other C21 steroids, relating to androgen and estrogen metabolism, appeared to be down-regulated with 1.3-21 fold decreased expression of multiple hydroxysteroid dehydrogenases (Hsd3b1, Hsd3b3, Hsd3b4, Hsd3b5, Hsd3b7, and Sdr42e1), and steroid-5-alpha-reductase (Srd5a1, -1.5 fold). Perturbations related to cholesterol and its derivatives might be associated with cellular stress responses further described below.

In both mouse strains, CMP013 treatment was associated with molecular evidences of reactive oxygen species (ROS) generation and an oxidative stress response in the cell. Aside from the induction of numerous phase 2 enzymes that had roles in eliminating ROS, Nrf2 also coordinated the transcriptional increases of antioxidant genes such as NAD(P)H dehydrogenase, quinone 1(Nqo1, 2.4 fold in WT, 1.7 fold in PXR-KO), and kelch-like ECH-associated protein 1 (Keap1, 1.4 fold in WT, 1.3 fold in PXR-KO). In the WT model, we further observed the transcriptional increases of glutathione peroxidase 2 (Gpx2, 3.5 fold), glutathione reductase (Gsr, 3.1 fold), thioredoxin (Txn, 1.4 fold), and DnaJ (Dnajb2, Dnajb6, Dnajb9, Dnajc2, 1.3-2 fold). In PXR-KO mice, increased expression was observed for thioredoxin reductase 1 (1.4 fold PXR-KO), heme oxygenase (Hmox1, 2.8 fold), flavin containing monooxygenase (Fmo1, 1.5 fold), peroxiredoxin 1 (Prdx1, 1.4 fold), and multiple DnaJ (a2, b1, b4, b6, c1, c2, c3, and c10, ∼1.5 fold). Furthermore, evidence for perturbations in the endoplasmic reticulum (ER) function was noted with the altered expression of genes signaling ER stress. In particular, we observed increased expression of eukaryotic translation initiation factor 2-alpha kinase 3 (Eif2ak3 or Perk, 1.4 fold in WT, 1.6 fold in PXR-KO), the ER stress sensor activating transcription factor 6 (Atf6, 1.3 in WT, 1.8 fold in PXR-KO), membrane-bound transcription factor peptidase, site 2 (Mbtps2 or S2p, 1.4 fold in WT, 1.7 fold in PXR-KO), and Mapk8 (1.6 fold WT, 1.7 fold in PXR-KO). These collective transcriptional changes suggested that such ER stress was associated with an accumulation of unfolded proteins. Cells responded by decreasing the rate of protein translation to prevent the accumulation of unfolded proteins (via the action of Perk and its target eukaryotic initiation factor 2, eIF2) and activating regulated intramembrane proteolysis [19] (via the action of Atf6 and S2p). There appears to be an interesting relationship between ER stress and increased cholesterol biosynthesis. Previously, Lee *et. al.* reported that ER stress inhibited the synthesis of Insig-1 protein, which in turns allowed Srebp proteolytic activation even in the presence of sterols [20]. The authors used cultured cells transfected with human Insig-1 and Insig-2 and herpes simplex virus Srebp2; here we were observed a similar phenomenon *in vivo*.

Lastly, there were perturbations in bile acid homeostasis associated with CMP013 exposure in both WT and PXR-KO models. Alteration to biliary transport was noted with increased expression of the canalicular transporters Abcb1 (or Mdr1, 3.8 fold), the basolateral transporter Abcc3 (or Mrp3, 2.4 fold), and the basolateral uptake transporter Slco1a2 (or Oatp, 11 fold). Increased expression of Mrp3 observed in the PXR-KO model suggested that another nuclear receptor was compensating for PXR in regulating bile acid homeostasis. In WT mice, we further observed increased expression of the canalicular transporter Abcc2 (Mrp2, 1.9 fold). In PXR-KO mice, Cyp7b1, encoding oxysterol 7alpha-hydroxylase, which converted cholesterol to bile acids, decreased 1.5 fold in expression. Cyp8b1, encoding the enzyme sterol 12-alpha-hydroxylase, which controls the balance between cholic acid and chenodeoxycholic acid secreted into the bile, decreased 1.9 fold in expression. These transcriptional changes indicated that CMP013 perturbed the transport and recycle of bile acids and their conjugates in WT mice and affected the biosynthesis and metabolism of bile acids in the knock-out strain. Together, the changes were suggestive of a cholestatic response induced by the compound [21].

## Discussion

The present study describes a novel algorithm for analyzing gene expression data and its application in studying the mechanism of toxicity of a drug candidate. Results with the proposed method were compared with analysis obtained with SAM to identify strengths and weaknesses associated with the new approach. First, a compelling aspect of our approach is that it is straightforward in its assumptions and therefore would be relatively easy for investigators to determine if the method is suitable for their experiments or questions. The threshold procedure in step 1 is simple and investigators can easily substitute an alternate cutoff that constitutes biological significance in their experiment. In the current study, by allowing a small cutoff on fold change, we increase our sensitivity for subtle expression changes because it is important, in toxicological assessment, to maintain a low false negative rate, even at the expense of false positives. This low threshold facilitates the discovery of signaling and regulatory genes which can produce a large downstream effect with a small change in expression. In fact, as most regulatory proteins are regulated by post-translational modification, only a small transcriptional increase is necessary to supplement the allosteric changes of the proteins. As compared to results obtained with SAM, the current method produced improved results in at least two pathways. For example, none of the genes in the ER stress response were found with SAM in WT mice. These genes have relatively small fold change (1.25 – 1.5), but the combined increase of multiple genes in this pathway strongly suggests that the ER stress response is not insignificant. The second example involves the cholesterol biosynthesis pathway in PXR-KO mice (Table 2). The current method identifies six genes while SAM only identifies three as differentially significant. Since pathway mapping by IPA is determined by the Fisher's Exact test, the presence or absence of these few genes may affect whether the pathway is considered activated. In looking at differentially expressed genes mapped to canonical pathways (Table 3), we noted that the overlap between results of the non-parametric method and SAM was much higher (∼70% as opposed to 45–55% in Figure 5). A possible explanation for this is that genes with annotation tend to be those that have been easily detected, resulting in their better characterization in the literature, and this robustness to measurement is also reflected in our study.

Inherent to the threshold approach in step 1 is that the current procedure accounts only for directional changes and disregards any differences in magnitude of changes as long as the fold change cutoff is satisfied. In other words, the method does not use the level of mRNA measured to determine if one pathway is more strongly up or down regulated than others. Our rationale for this is two fold. First, since the levels of mRNA fluctuate rapidly, it is usually not possible to make an accurate determination of the level of induction based on measurements at limited time points. That is, beyond a threshold of fold change used to signify biological difference, information on the magnitude of expression change is likely to be highly dependent on the exact time of measurement and therefore is not a reliable indicator of relative level of pathway activation. Second, even when such ranking of pathway activation can be determined, this information does not always improve the overall understanding of the underlying biology since only qualitative information about pathway modulation, i.e., whether a pathway is up- or down-regulated, is transferable across experiments. Alternative to the magnitude of change, we believe that the statistical significance of modulation can be more reliably estimated based on the consistency and similarity of responses from animals in the same cohort. Accordingly, perhaps a disadvantage of this method is that it necessitates at least five animals per treatment group and does not perform well with small group sizes. Similar to SAM and many other non-parametric statistics, the null distribution is created by permuting sample labels. Given an experimental design of two groups, sample permutation of group size of four produces maximally only 70 different possibilities, and this number, from our experience, is too small to generate a sufficiently informative null distribution for FDR estimation. In the drug development setting, the requirement of group size larger than three makes the method somewhat impractical for many screening studies where the group size may be limited to three, given the large number of drug candidates and the intent for these studies. However, for most investigational and mechanism identification studies, the number of animals per group tends to be larger and thus more suitable for this method.

Finally, perhaps another undesirable aspect of the threshold procedure is that it does not deal well with sequences which show increased and decreased expression in equal number of animals.

In fact, we have resolved these situations by pre-filtering out all such genes from consideration as differentially expressed (Materials & Methods section). With methods in which absolute levels of expression are evaluated, it is possible that the genes could be considered differentially expressed if the magnitude of change in one direction far exceeds that in the other direction. In fact, looking at genes that were only identified by SAM, we found approximately 100 genes falling in this category (extreme ends of Figure 5DF). These genes in vehicle-treated animals showed mixed increased and decreased expression relative in the group mean, but the genes were identified as differentially expressed because the inter-group difference far exceeds intra-group difference. We understand that failure to discover these genes is perhaps a drawback of the current method. However, given the uncertainty associated with magnitude of expression changes as discussed above, it is unlikely that all of these genes are associated with true signals. It is noteworthy to mention that if such genes were associated with a true signal, one may still be able to realize the associated biological response by the expression of other genes involved in the same pathway. We therefore feel that the elimination of such genes does not necessarily impact our overall interpretation of the experiment. Our best recommendation in these situations would be to apply our analysis method in conjunction with an algorithm similar to SAM, i.e. using two methods that have complementary approaches, so that one can obtain a clear picture of the overall transcriptional response.

In summary, we describe a novel non-parametric statistical method for the analysis of gene expression data for studies in which conventional variance-based analysis methods result in suboptimal results. Indeed, the benefit of our method is substantiated for datasets from preclinical or clinical studies where subject-to-subject variations are relatively large. The method is straightforward in its assumptions and allows investigators to specify criteria for both biological significance and statistical significance. In the mouse-knockout example described here, the application of this method allowed us to unravel the molecular mechanisms associated with hepatic toxicities induced by an inhibitor of β-secretase in the presence and absence of the nuclear hormone receptor PXR.

## Supporting Information

Data S1 Differentially expressed genes resulting from CMP013 treatment in wild type and knockout mice.
(XLS)
